# Activated Natural Killer Cells Hit Neurogenesis in the Aging Brain

**DOI:** 10.1007/s12264-021-00654-3

**Published:** 2021-03-29

**Authors:** Angelica Cuapio, Hans-Gustaf Ljunggren

**Affiliations:** Center of Infectious Medicine, Department of Medicine Huddinge, Karolinska Institutet, 141 52 Stockholm, Sweden

Why neurogenesis is attenuated in elderly individuals is an intriguing question that has raised renewed interest. Mechanisms associated with declined neurogenesis in the aged brain have been attributed to inflammatory cytokines [1]. More recently, a specific role for interferon-γ (IFN-γ) produced by CD8-expressing cytotoxic T cells has been implicated [2]. These observations suggest a scenario in which neurogenesis, at least in part, is regulated by immune cells within the aging brain. This raises several interesting questions with regards to the characteristics of specific immune cells within the brain, the signals for their expansion and maintenance, and their role in affecting neurogenesis and cognition during normal brain aging.

Further detailed insights into these processes have now been provided. In a recently published study [3], Wei-Na Jin *et al*. analyzed the subtypes, frequencies, and location of immune cells in young and aged brains. Strikingly, an abundant population of natural killer (NK) cells in the dentate gyrus of brains from old humans was observed. NK cells are innate lymphocytes, with some adaptive features, that normally play a critical role in fighting virus infections and tumors [4]. These NK cells outnumbered neutrophils, monocytes, and adaptive T and B lymphocytes in the brain, and were characterized by the expression of specific activation and cytotoxicity markers. These and other observations led the authors to conclude that NK cells may accumulate in specific regions of the human brain with age, in particular in the dentate gyrus. Similar observations were made in mouse studies. To gain a more detailed understanding of the effects of aging on the immune landscape in the dentate gyrus, the authors performed single-cell RNA sequencing-analysis of immune cells obtained from dentate gyrus tissues of young and aged mice. Dentate gyrus-specific alterations in NK cells were found for molecules related to cytotoxicity, target cell specificity, tissue retention, and cytokine-mediated signaling. Flow cytometric analysis corroborated these studies, revealing that CD69 (an activation and tissue residency marker), NKG2D (an activating cell surface receptor), and perforin and granzyme B (cytotoxicity mediators) were markedly increased in NK cells from the aged dentate gyrus.

**Fig. 1:**
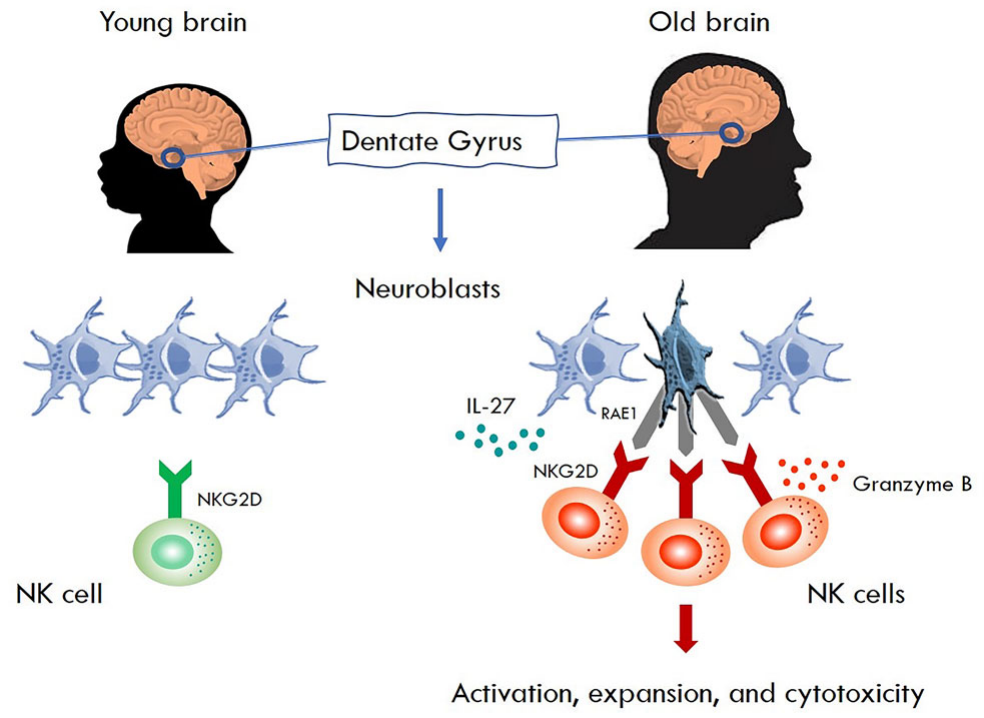
NK cells impair neurogenesis in the old brain.

During organ inflammation, NK cells are often recruited to the target tissue [5]. To determine if NK cells in the dentate gyrus were recruited from the periphery or expanded locally, the authors tracked NK cells using advanced parabiosis experiments, conjoining the vasculature of a mouse where all NK cells are labeled with the fluorescent protein tdTomato with that of a normal mouse. In such experiments, few NK cells accumulated in the dentate gyrus of parabiotic wild type mice, suggesting that recruitment may have a limited contribution to NK cell accumulation in the aged dentate gyrus. These studies, together with additional fate mapping analyses, provided further support for local expansion and accumulation of NK cells in the aged dentate gyrus. Furthermore, the authors found that the NK cell chemokine CCL3 and the growth factors GM-CSF, IL-2 and, particularly, IL-27 were produced in relatively high amounts in the interstitial fluid of the aged dentate gyrus. In studies determining whether IL-27 derived from aged neuroblasts was necessary for local expansion and accumulation of NK cells in the aged dentate gyrus, it was observed that an IL-27-neutralizing antibody blocked these effects. Together, these and other results suggested to the authors that neuroblasts sustain NK cells and augment their cytotoxicity in the aged dentate gyrus mediated, at least in part, via IL-27 (Fig. 1).

In the brain, NK cells increase with age. They predominantly reside in the dentate gyrus, a neurogenic niche where neuroblasts are also found. As the brain ages, neuroblasts undergo cellular senescence, start to express RAE-1, and produce high levels of IL-27 that induces expansion of NK cells. Notably, RAE-1, as depicted in the figure, is a ligand for the NK cell activation receptor NKG2D. These age-related alterations trigger cytotoxic activity by the NK cells leading to loss of neuroblasts, concomitantly preventing regeneration of neurons resulting in cognitive decline.

The study of Wei-Na Jin *et al*. also provides new evidence with respect to cellular senescence, and how it may contribute to neuroinflammation in the aging brain. Transcriptomic analyses revealed that aging induced a repression of cell cycle genes and an upregulation of senescence-associated secretory phenotype (SASP) genes in neuroblasts. To more directly address the possible involvement of NK cells in neurogenic decline, the authors temporarily depleted NK cells from old mice. Strikingly, in aged mice depleted of NK cells, they found an increase of dentate gyrus neuroblasts but not neural stem cells. Additional studies revealed reduced counts of caspase-3^+^ neuroblasts in the aged dentate gyrus of mice depleted of NK cells providing support for an NK cell-mediated contribution to neuroblast apoptosis. These and other findings were corroborated by *in vitro* NK cell lysis studies, in which the authors observed that NK cells could target aged neuroblasts. Together, these results revealed strong evidence for NK cell-mediated elimination of aged dentate gyrus neuroblasts.

Seeking a molecular mechanism for NK cell-recognition of aging neuroblasts, Wei-Na Jin* et al*. identified an upregulation of NK cell activating ligands, predominantly retinoic acid early inducible 1 (RAE-1), an NKG2D-activating receptor ligand that can trigger NK cell activation and cytotoxicity (Fig. 1). Notably, mTOR inhibition diminished the upregulation of RAE-1 in the aged neuroblasts. Furthermore, blockade of RAE-1-NKG2D interactions protected neuroblasts against NK cell lysis. Finally, having found that NK cells eliminate neuroblasts in the aged dentate gyrus, the authors reasoned that NK cells might contribute to age-related decline in cognition and synaptic plasticity. For this purpose, they examined spatial learning after NK cell removal. Upon depletion of NK cells in aged mice, they found improved spatial learning performance. These and other results led Wei-Na Jin and collaborators to suggest that NK cells may impair cognition and hippocampal synaptic plasticity, and that this may occur independent of adaptive B and T cells of the immune system.

Interestingly, the study by Wei-Na Jin *et al*. bears some resemblance to studies of NK cells targeting specific neuronal cells within the peripheral nervous system (PNS). In early studies of NK cell-mediated lysis of dorsal root ganglia (DRG) neurons *in vitro*, interactions of the NK cell activating-receptor NKG2D with the endogenous ligand RAE-1 expressed by DRG cells triggered NK cell lysis [6, 7]. Corroborating these early studies, a recent study showed that NK cells expressing NKG2D infiltrates damaged peripheral nerves in mice [8]. Here, also the expression of RAE-1 contributed to cytotoxicity that correlated with loss of sensation due to degeneration of injured afferent axons. Whether similar processes operate also in humans, and in the context of other neurological diseases such as stroke, dementia, Parkinson´s disease, Alzheimer´s disease or even Covid-19 related neurological symptoms, may constitute future avenues of research.

The understanding of inflammaging, a chronic non-infectious and low-grade inflammatory process predominantly associated with age-related metabolic diseases, has increased significantly in recent years [9]. Inflammaging in the brain has been less well studied. The brain has historically been considered as an immune-privileged organ as it shows a low permeability of immune cells [10]. However, in response to infection, lymphocyte recruitment and local inflammatory processes in the brain are observed. If low grade immune cell influx to the brain tissue also occurs during normal physiological, non-infectious, processes is less clear. Recently, there has been an increased interest in tissue resident NK cells (trNK) and their function not only in mounting classical immune responses, but also in tissue development and remodeling [11]. Particularly, in the brain tissue, knowledge has largely been lacking with respect to the existence of tissue resident lymphocyte populations including NK cells. The present study not only sheds new light onto mechanisms controlling neurogenesis in the human brain, but also reveals new information on the possibility that certain anatomical areas of the aging brain may constitute yet a novel niche of possible “tissue specific” NK cells. Furthermore, it is remarkable that T cell-secreted IFN-γ [12] has been related to decreased proliferation of neural stem cells [2]. Since NK cells are prominent producers of IFN-γ [4, 12], it is tempting to speculate that NK cell IFN-γ may exert a similar function in the brain.

Noteworthy, while the present study has focused on NK cells in the dentate gyrus, other types of cells might also affect neurogenesis in this particular location. The latter include resident microglia, the principal immune cells of the brain. Although microglial activation has a deleterious role in ischemic stroke and its suppression is considered as a promising therapeutic target, recent studies also consider microglial activation in context of being critical not only for neurogenesis, but also for synaptic remodeling and angiogenesis (as reviewed in [13]). Complementary research on the role of microglia in the dentate gyrus of adult human brain will help to understand the complexity of microglia-NK cell interaction during aging.

In analogy to the connectome, where connections from neurons and synapses are deciphered to understand the organization of neural interactions in the human brain, the study of the interactions between components of the immune system and the nervous system is vital to map interrelations associated with aging as well as to specific neurological diseases. The present study provides us with pioneering glimpses of the anatomical location and function of NK cells in the brain, highlighting an NK cell-dependent role in age-related decline of neurogenesis and cognition. However, it still needs to be investigated if the findings of the present study, as well as related studies on T cell infiltration in neurogenic niches of the mouse brain, translate to the human situation. It would be fascinating to investigate whether these discoveries also bare relevance to a variety of other age-related disorders accompanied with impaired tissue regeneration. Future studies could cover possible therapies designed to target the immune system, or tissue specific immune reactions, as a way of combating age-related regenerative and cognitive dysfunctions.
